# Enhanced thermal and alkaline stability of L-lysine decarboxylase CadA by combining directed evolution and computation-guided virtual screening

**DOI:** 10.1186/s40643-022-00510-w

**Published:** 2022-03-21

**Authors:** Yang Xi, Lidan Ye, Hongwei Yu

**Affiliations:** 1 Institute of Bioengineering, College of Chemical and Biological Engineering, Zhejiang University, Hangzhou, 310027 China; 2 Hangzhou Global Scientific and Technological Innovation Center, Zhejiang University, Hangzhou, 311200 China

**Keywords:** Cadaverine, L-lysine decarboxylase, Stability, Directed evolution, Virtual screening

## Abstract

As an important monomer for bio-based nylons PA5X, cadaverine is mainly produced by enzymatic decarboxylation of L-lysine. A key issue with this process is the instability of L-lysine decarboxylase (CadA) during the reaction due to the dissociation of CadA subunits with the accumulation of alkaline cadaverine. In this work, we attempted to improve the thermal and alkaline stability of CadA by combining directed evolution and computation-guided virtual screening. Interestingly, site 477 residue located at the protein surface and not the decamer interface was found as a hotspot in directed evolution. By combinatorial mutagenesis of the positive mutations obtained by directed evolution and virtual screening with the previously reported T88S mutation, K477R/E445Q/T88S/F102V was generated as the best mutant, delivering 37% improvement of cadaverine yield at 50 ºC and pH 8.0. Molecular dynamics simulations suggested the improved rigidity of regional structures, increased number of salt bridges, and enhancement of hydrogen bonds at the multimeric interface as possible origins of the improved stability of the mutant. Using this four-point mutant, 160.7 g/L of cadaverine was produced from 2.0 M Lysine hydrochloride at 50 °C without pH regulation, with a conversion of 78.5%, whereas the wild type produced 143.7 g/L cadaverine, corresponding to 70% conversion. This work shows the combination of directed evolution and virtual screening as an efficient protein engineering strategy.

**Graphical Abstract:**

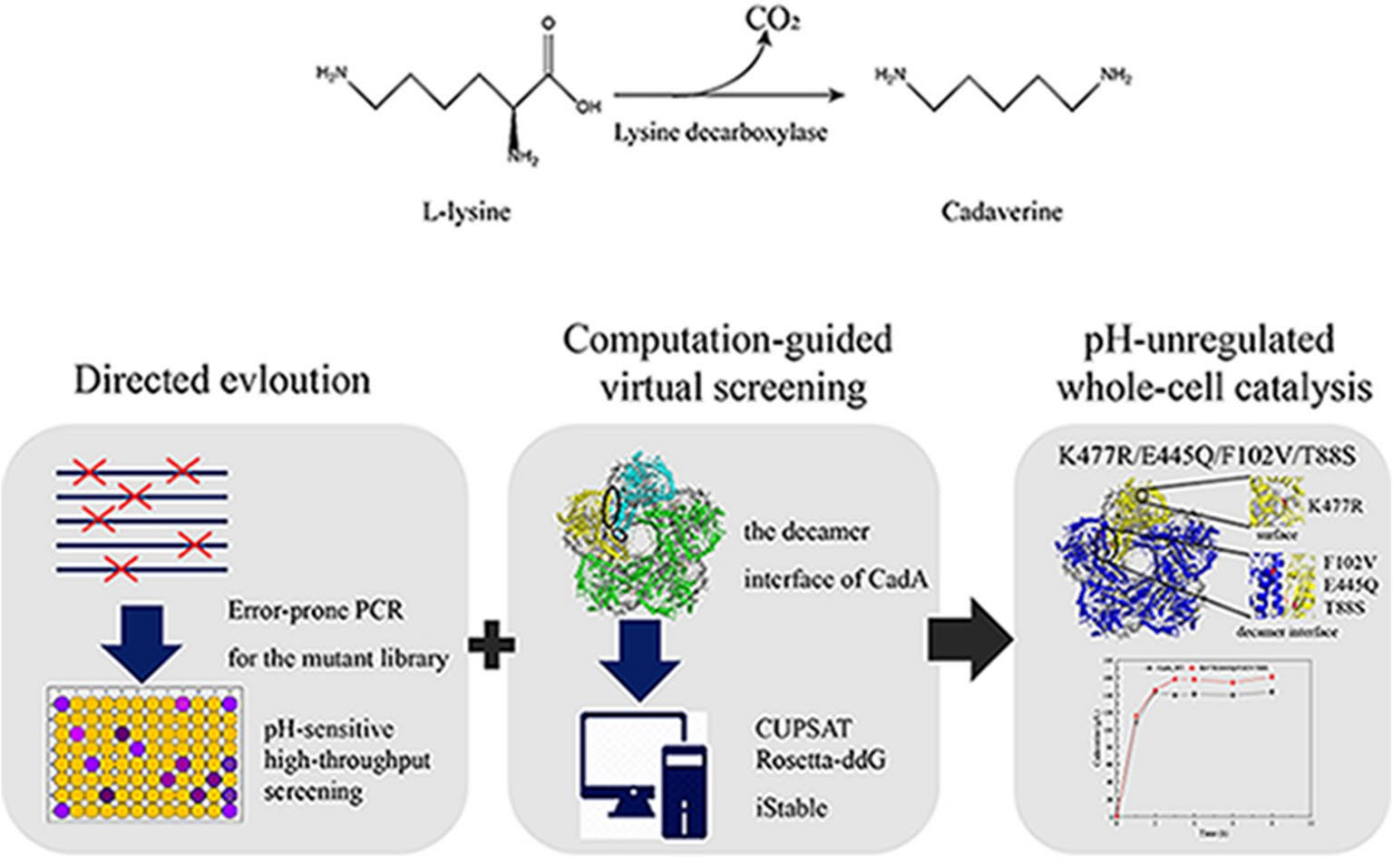

**Supplementary Information:**

The online version contains supplementary material available at 10.1186/s40643-022-00510-w.

## Introduction

As an important raw material of bio-based nylons PA5X and chelating agents, cadaverine has great commercial values (Kind and Wittmann 2011; Ma et al. 2017). At present, whole-cell catalysis is the main approach to producing cadaverine, and the production efficiency is mainly limited by the catalytic performance of L-lysine decarboxylase, which is a pyridoxal-5-phosphate (PLP)-dependent enzyme. L-lysine decarboxylases are found in a wide range of microorganisms, such as *Escherichia coli*, *Bacterium cadaveris*, *Aliivibrio salmonicida*, *Hafnia alvei*, *Selenomonas ruminantium* (Fecker et al. 1986; Schneider and Wendisch 2011; Wang et al. 2015; Jeong et al. 2016; Kou et al. 2016), among which CadA from *E. coli* is widely used to produce cadaverine in the industry owing to its high catalytic activity and protein expression level (Kim et al. 2015a; Leong et al. 2020). However, CadA only has a high catalytic activity in the pH range of 5.5–6.5 (Lemonnier and Lane 1998; Kanjee et al. 2011), which conflicts with the alkaline nature of cadaverine. With the accumulation of alkaline cadaverine during the reaction, dissociation of CadA subunits occurs, causing inactivation of the enzyme (Watson et al. 1992; Kanjee et al. 2011). Therefore, a large amount of acid should be added to maintain a relatively low pH during cadaverine production, followed by addition of massive alkali to separate cadaverine in the downstream process, which increases the costs and causes environmental burden (Kind et al. 2014; Ma et al. 2017). Meanwhile, a high reaction temperature can increase substrate solubility and accelerate the reaction. Therefore, a CadA mutant with improved thermal and alkaline pH stability would be of great significance for large-scale industrial production of cadaverine.

Previous efforts have been made to improve the stability of CadA, mostly by means of rational design targeting at the residues on the subunit interface. For instance, a double mutant F14C/K44C showing improved thermal and alkaline pH stability was created by introducing disulfide bonds in the multimeric interface of CadA; however, the catalytic activity was decreased (Hong et al. 2017). In another study, mutant T88S was created by computational saturation mutagenesis at the T88 position located in CadA decamer interface, showing improvement in both stability and catalytic activity (Kou et al. 2018). Despite these progresses in CadA engineering, exclusive selection of the interface residues as the engineering targets has limitations. Due to the complex three-dimensional structures of enzymes, the introduction of mutations at other regions of the protein may also affect the structure of the multimeric interface, thus affecting protein stability (Reetz et al. 2009; Yu and Dalby 2018). Directed evolution which works through screening of mutant library generated by random mutagenesis often leads to discovery of mutagenesis hot spots in distant residues (Sheldon and Pereira 2017; Arnold 2018). Meanwhile, algorithms and software for prediction of protein stability have emerged with the rapid development of computer technology, such as Rosetta_ddG, CUPSAT, iStable, FoldX, and Fireport, which largely saves experimental workloads and contributes to construction of small-size mutant libraries with high quality (Wijma et al. 2014; Bednar et al. 2015; Arabnejad et al. 2017; Romero-Rivera et al. 2017). Therefore, combination of directed evolution and virtual screening may serve as an efficient strategy for improving protein stability.

In this study, *E. coli* CadA was engineered for improved thermal and alkaline pH stability by combining directed evolution and computation-guided virtual screening. First of all, a high-throughput screening method is developed for distinguishing lysine decarboxylase activity at 50 ºC and pH 8.0. Subsequently, directed evolution of CadA is conducted based on this high-throughput screening method. Meanwhile, virtual saturation mutagenesis of residues on the decamer interface of CadA is performed using iStable, CUPSAT, and Rosetta-ddG, respectively. Finally, the positive mutations selected out by the above methods are combined to generate the best mutant, and the possible mechanism behind the stability improvement is discussed via molecular dynamics simulations.

## Materials and methods

### Strains, plasmids, and chemicals

pET-30a(+) was used as the expression vector. *E. coli* strains BL21(DE3) and K12 were used for DNA transformation and gene cloning, respectively. *EasyTaq* DNA polymerase and *FastPfu Fly* DNA polymerase were purchased from TransGen Biotech (Beijing, China). PrimeSTAR HS DNA polymerase, *Bam*H I, *Hind* III, and T4 DNA ligase were purchased from Takara (Dalian, China). L-lysine hydrochloride was purchased from Macklin (Shanghai, China). PLP was purchased from Sangon Biotech (Shanghai, China). Other chemicals were all of analytical grade purity.

### Mutant library construction

The *cadA* gene was cloned into pET-30a(+) and then used as the template for error-prone PCR to construct the mutation library of CadA. The primers used are listed in the supplementary material (Additional file 1: Table S1). The error-prone PCR reaction mixture contained 10 × *EasyTaq* buffer (2.5 μL), ddH_2_O (16.0 μL), dNTPs (2.5 mM, 2 μL), Mn^2+^ (1 mM, 2.5 μL), template plasmid (0.5 μL), forward and reverse primers (10 μM, 0.5 μL each), and *EasyTaq* DNA polymerase (2.5 U). The PCR program was 3 min denaturation at 94 ºC, followed by 30 cycles of 95 ºC for 30 s, 55 ºC for 30 s, 72 ºC for 140 s, and 10 min final elongation at 72 ºC.

The error-prone PCR products were used as primers to generate mutation-containing plasmids by MEGAWHOP (megaprimer PCR of whole plasmid) with pET-30(a)-cadA as the template. The MEGAWHOP reaction mixture was composed of the template plasmid (0.5 μL), 5 × *FastPfu Fly* DNA buffer (2.5 μL), Mg^2+^ (50 mM, 0.5 μL), dNTPs (2.5 mM, 2 μL), PCR stimulant (2.5 μL), ddH_2_O (13.0 μL), megaprimer (1 μL), and *FastPfu Fly* DNA polymerase (0.5 μL). The MEGAWHOP program consisted of a denaturation step at 95 °C for 2 min, followed by 25 cycles of denaturation at 95 °C for 20 s, annealing at 55 °C for 20 s, and elongation at 72 °C for 230 s. The final elongation was performed at 72 °C for 5 min. The PCR product was digested by the DMT enzyme (TransGen Biotech) at 37 ºC for 2 h and then transformed into competent cells of *E. coli* BL21 (DE3). Finally, the cells were incubated overnight at 37 °C on the LB agar plates supplemented with 50 μg/mL of kanamycin.

### Library screening

Individual colonies of the CadA mutant library were selected into 96 deep-well plates containing 300 μL LB media supplemented with 50 μg/mL of kanamycin. After overnight incubation at 37 °C and 220 rpm, 10 μL of seed cultures were inoculated into a new 96 deep-well plate containing 500 μL of LB media supplemented with 50 μg/mL of kanamycin. After incubation at 37 °C and 220 rpm for 2 h, isopropyl-β-thiogalactopyranoside (IPTG) was added to a final concentration of 0.1 mM and incubated at 25 °C and 220 rpm for another 10 h to induce enzyme expression. The cells were harvested by 10 min centrifugation at 4000 rpm and 4 ºC, followed by washing twice using 0.9% saline solution.

The cells in each well were suspended in 500 μL borate buffer (5 mM, pH 8.0). The cell suspension (10 μL) was transferred into 96 deep-well plates, and 490 μL of substrate buffer (100 mM L-lysine hydrochloride, 0.1 mM PLP in 5 mM borate buffer, pH 8.0) was added. The reaction plates were incubated at 50 °C and 220 rpm for 30 min. The reaction was stopped at 100 ºC for 5 min, and centrifuged at 4000 rpm for 10 min to remove the cells. The reaction mixture (150 μL) was then transferred into new 96-well microtiter plates, together with 20 μL of the mixed indicator composed of 0.1% thymol blue and 0.1% phenolphthalein (3:1, v/v). The absorbance of the reaction mixture was measured at 550 nm by EPOCH2 (BioTek, USA). The mutants with improved activity at 50 ºC and pH 8.0 as indicated by the higher absorbance were selected out.

### Computational saturation mutagenesis

The crystal structure of CadA (PDB Number: 3N75) was obtained from the PDB database (https://www1.rcsb.org/). Computational saturation mutagenesis was conducted by CUPSAT (Cologne University Protein Stability Analysis Tool), iStable and Rosetta_ddG, respectively. CUPSAT is a web tool to analyze and predict protein stability of single amino acid mutations based on structural environment specific atom potentials and torsion (Parthiban et al. 2006). The PDB Number was input to CUPSAT (http://cupsat.uni-koeln.de/) to predict the difference in free energy of unfolding between the wild type and the single-point mutants using specific atom potentials and torsion angle potentials. iStable (http://predictor.nchu.edu.tw/iStable/) is an integrated prediction tools with grid computing architecture constructed by prediction results from different element predictors (Chen et al. 2013, 2020). The PDB number, temperature, and pH were input to iStable to predict the change in the stability of a single-point mutant. The changes in folding free energy △△G (△△G = △G_MUT_ –△G_WT_) of the CadA variants were calculated using Rosetta Cartesian_ddG, substituting with all proteinogenic amino acids except cysteine (Arabnejad et al. 2017). △△G < -1.0 kJ/(mol·subunit) was defined as a mutant with improved stability.

### Analytical methods

L-lysine hydrochloride and cadaverine were derivatized with diethyl ethoxymethylenemalonate (DEEMM) before quantification (Kim et al. 2015b). The DEEMM derivatization mixture contained 80 μL of 10 mM sample, 32 μL of 200 mM DEEMM, 480 μL of borate buffer (50 mM, pH 9.0), 160 μL ethanol, and 48 μL ddH_2_O. To remove the excessive DEEMM, the derivatization mixture was incubated at 70 ºC for 2 h. The derivatized samples were quantified on a Shimadzu HPLC system (LC-20A) equipped with a ZORBAX Extend-C18 (4.6 × 150 mm) column (Agilent, USA) and a UV/Vis detector. The mobile phase was composed of sodium acetate buffer (25 mM, pH 4.8) (A) and 100% acetonitrile (B). The samples were eluted at a flow rate of 1 mL/min with a gradient program: 0–2 min, 20–25% A; 2–32 min, 25–60% A; 32–40 min, 60–20% A. The signals were detected at 284 nm.

### Enzyme purification and activity assay

The *E. coli* cells expressing the wild type and mutants of CadA were collected and re-suspended in binding buffer (20 mM phosphate buffer, 0.1 mM PLP, 0.1 mM dithiothreitol (DTT), 20 mM imidazole, 0.5 mM NaCl) to 50 g wet cells per liter and disrupted by sonication. The supernatant containing crude enzyme was collected by centrifugation at 4000 rpm and 4 °C for 20 min. After filtration through a 0.45 μm filter, the enzyme was purified using a Ni-NAT column (Sangon Biotech, Shanghai) and desalted using a DS-10 G-25 column (Geochrom Biological, Wuhan). The purity of the proteins was determined by SDS-PAGE.

Protein concentrations were determined with the BCA Protein Assay Kit (Sangon Biotech, Shanghai). The activity of lysine decarboxylase was tested using Phan’s method (Phan et al. 1982), based on the different solubilities of products formed from 2,4,6-trinitrobenzenesulfonic acid (TNBS) and lysine or cadaverine. N,N’-bisnitrophenylcadaverine (TNP-cadaverine) was extracted by toluene, and the absorbance at 340 nm was measured. One unit of enzyme activity was defined as the amount of enzyme producing 1 μmol cadaverine per minute at 37 ºC.

### Molecular dynamics simulations

The crystal structure of the wild-type CadA was obtained from the protein databank (PDB Number: 3N75). The models of the mutants were constructed by Chimera. The ligand model was built using ChemOffice 2014. Substrate molecular dockings were performed using AutoDock 4. Molecular dynamics simulations were conducted using AmberTools 18. Counterions of Na^+^ were added to neutralize the system, which was filled with TIP3P water molecules under periodic boundary conditions (Yang et al. 2017).

### Whole-cell catalysis

The whole-cell reaction was performed at 50 °C and pH 6.0. The reaction mixture contained 2.0 M L-lysine hydrochloride, 0.1 mM PLP, 6.0 g wet cells per liter of whole-cell biocatalyst, and acetate buffer (500 mM, pH 6.0). No extra acidic solutions were added to regulate the pH of the reaction mixture during the reaction.

### Statistical analysis method

Statistical significance of the different data in comparison with the wild type was evaluated using Student’s *t* test (*, *P* $<$ 0.05, **, *P* $<$ 0.01).

## Results and discussions

### Development and validation of the high-throughput screening method

**Fig. 1:**
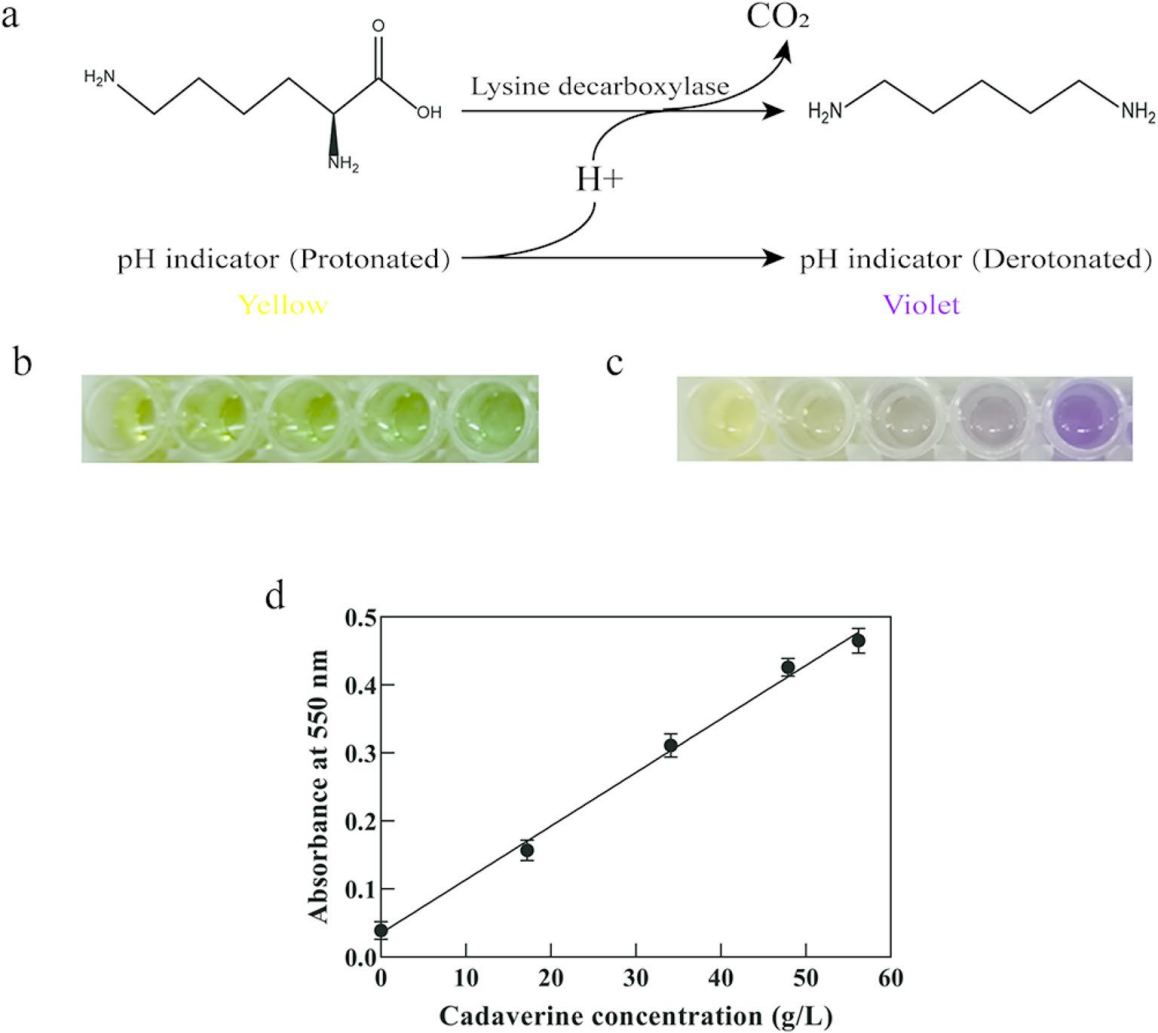
Establishment of the HTS method. **a** Colorimetric method for the determination of lysine decarboxylase activity. **b** Color change of thymol blue with pH increase at pH 8.0. **c** Color change of the mixed indicator with pH increase at pH 8.0. **d** Correlation between the absorbance at 550 nm and the HPLC results. The reaction was conducted at 50 ºC in borate buffer (pH 8.0), and samples were taken after 0, 15, 30, 45, and 60 min of reaction. The cadaverine concentration was determined by HPLC. The error bars represent standard deviations calculated from triplicate experiments

In order to obtain lysine decarboxylase mutants with improved stability under alkaline and high-temperature conditions, a high-throughput screening (HTS) method was developed to select out mutants with higher catalytic activity at 50 ºC and pH 8.0. The principle of the HTS method is illustrated in Fig. 1a. With the accumulation of alkaline cadaverine produced by decarboxylation of lysine, the pH of the reaction system would increase. Therefore, the activity of lysine decarboxylase could be visualized by color changes of appropriate pH indicators.

When choosing pH indicators, the following factors should be taken into consideration. To ensure that the color change of the pH indicator is proportional to the number of protons consumed in the decarboxylation reaction, the p*K*a value of the buffer should be equal or similar to the p*K*a value of the indicator, and the color change point of the indicator should be compatible with the target reaction (Yu et al. 2011; Jiang et al. 2017). Based on these principles, thymol blue with a p*K*a value of 8.9 and color profile of yellow (pH 8.0) to blue (pH 9.6) and borate buffer with a p*K*a value of 8.21 and a buffered pH range from 8.0 to 10.0 were chosen for assay at an initial pH of 8.0. However, an illegible transition color (green) with a weak change of absorbance reduced the accuracy of screening (Fig. 1b). Additionally, in actual condition, the final pH of the decarboxylation reaction could not reach 9.6, so thymol blue seemed to be not the perfect indicator for mutant screening. In comparison, mixed indicators have superior properties, such as narrower color profiles, and are void of illegible transition colors. The mixed indicator composed of 0.1% thymol blue and 0.1% phenolphthalein (3:1, v/v) has a color change point at pH 9.0 and a color profile of yellow (pH 8.7) to violet (pH 9.3) (Fig. 1c). Therefore, it was selected for establishment of the HTS method.

To find out a suitable detection wavelength for determination of the color change during HTS, full-wavelength scanning was conducted for the protonated and deprotonated forms of the mixed pH indicator using an EPOCH2 microplate reader (BioTech, USA). As shown in Additional file 1: Fig. S1a, the wavelength with maximal difference in absorbance was 550 nm.

To validate the HTS method, different concentrations of NaOH were added to simulate the consumption of protons in the decarboxylation reaction, and the relationship between absorbance at 550 nm and proton consumption was examined. As shown in Additional file 1: Fig. S1b, the concentrations of borate buffer showed an important effect. In the concentration range of 5–20 mM, the higher the buffer concentrations, the broader the linear range and the lower the sensitivity. To ensure relatively high sensitivity and appropriate linear range of the HTS method, 5 mM borate buffer was selected.

Finally, the effectiveness of the HTS method was verified by HPLC analysis (Fig. 1d). The linear relationship between the cadaverine concentration and the absorbance at 550 nm demonstrated that the HTS method was suitable for directed evolution of CadA toward higher activity at 50 ºC and pH 8.0.

### Directed evolution of CadA for improved stability

**Fig. 2:**
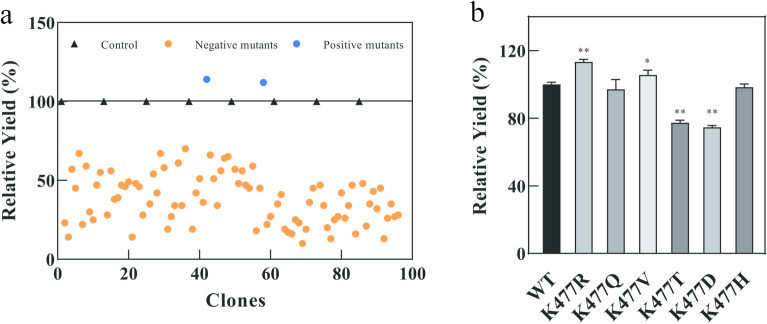
Directed evolution of CadA. **a** High-throughput screening of error-prone PCR mutation libraries. **b** Site-directed mutagenesis of K477. The error bars represent standard deviations calculated from triplicate experiments, and statistical significance of the different relative cadaverine yield in comparison with the wild type was evaluated using Student’s *t* test (*, *P* $<$ 0.05, **, *P* $<$ 0.01)

As shown in Fig. 2a, a majority of mutants generated by error-prone PCR were deleterious with much lower enzyme activity than the wild type, only a few strains showed improved enzyme activity. Finally, from the mutant library with a size of around 3000, two positive mutants K477R and F102V were screened out using the HTS method established above, with 15% and 10% improvement in cadaverine yield at 50 °C and pH 8.0, respectively.

Site 102 is located on the decamer interface of CadA and had been selected as an engineering target for stability improvement in a previous rational design effort (Hong et al. 2017). Surprisingly, site 477 as the other mutation hotspot identified in directed evolution is not located on the decamer interface but elsewhere at the surface of CadA, and it is therefore not a typical site that would be selected in rational engineering for altering the stability of CadA. The K477 site was further mutated into different types of amino acids to find the best substitution, including the polar amino acids Q and T, the non-polar hydrophobic amino acid V, the acidic amino acid D, and the alkaline amino acid H. However, they all showed lower cadaverine yield than K477R (Fig. 2b). Among the above-mentioned amino acids, only arginine (R) had a higher pI value than lysine (K) (10.76 vs 9.6), so the substitution of R for K could introduce more positive charges around the 477 site at pH 8.0. This result implied that increasing the positive charges on the CadA surface may have a positive effect on its catalytic performance under alkaline conditions.

### Computational saturation mutagenesis of selected sites on the decamer interface of CadA

**Fig. 3:**
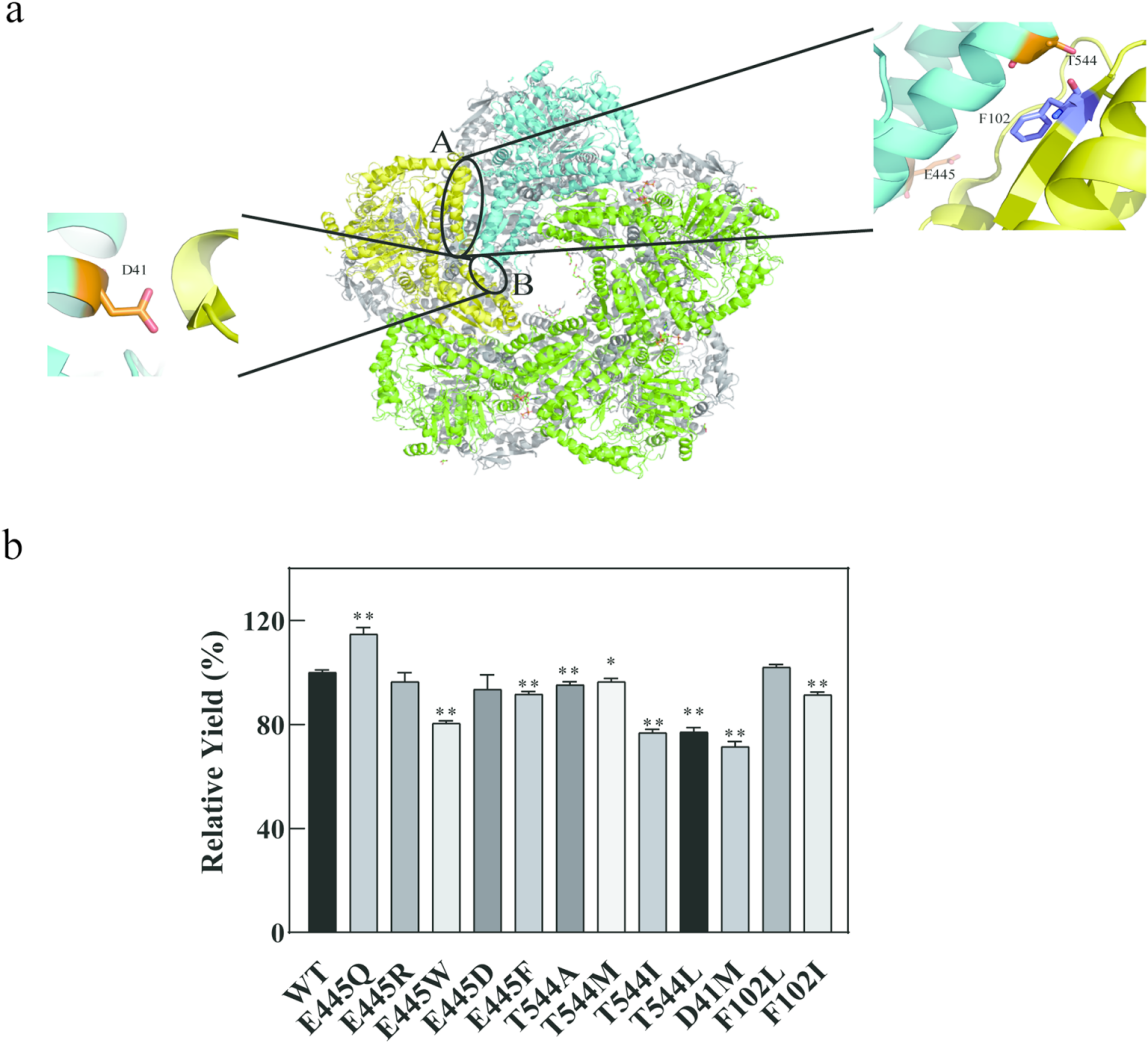
Computational saturation mutagenesis of the decamer interface of CadA. **a** The decamer interface region of CadA is a decamer composed of five dimers. F102, E445, and T544 are located in interfacial region A, while D41 is located in interfacial region B. **b** Experimental verification results of the mutants with improved stability as predicted by virtual screening. The error bars represent standard deviations calculated from triplicate experiments, and statistical significance of the different relative cadaverine yield in comparison with the wild type was evaluated using Student’s *t* test (*, *P* $<$ 0.05, **, *P* $<$ 0.01)

Dissociation of CadA subunits occurs with the accumulation of alkaline cadaverine, causing inactivation of the enzyme. Therefore, residues located on the decamer interface of CadA are important targets for improving the stability of CadA. In a previous report, disulfide bonds were introduced to sites 41, 102, 445, and 544 located on the decamer interface of CadA by amino acid substitution to obtain high-stability variants (Hong et al. 2017). Although introducing disulfide bonds is a common and effective method to improve protein stability (Eijsink et al. 2004; Badieyan et al. 2012), it may have negative impacts on enzyme activity. Most of the mutants generated by this strategy lost 60%–90% of the initial enzyme activity (Hong et al. 2017). In our study, virtual saturation mutagenesis was conducted for the above-mentioned sites instead using CUPSAT, iStable, and Rosetta-ddG, respectively (Fig. 3a). The prediction results are shown in the supplementary material (Additional file 1: Table S2). Experimental verification was conducted for the candidate mutants predicted as positive by two algorithms or more. The mutant E445Q was screened out with 13% improvement in cadaverine yield at 50 ºC and pH 8.0. Most of the other mutants had no significant improvement in the catalytic performance, and some of them even showed obviously decreased yield of cadaverine (Fig. 3b). Although the above-mentioned algorithms had been successfully used to enhance the stability of a number of proteins, such as ω-transaminase (Meng et al. 2020) and lipase (Li et al. 2018), they seemed to be not very accurate in prediction of the decamer CadA with relatively complex structure.

### Combinatorial mutagenesis

Based on the three positive mutations K477R, F102V, and E445Q identified in directed evolution and virtual screening, together with the previously reported T88S mutation (Kou et al. 2018), combinatorial mutagenesis was conducted. As shown in Table 1, among the double mutants constructed based on the K477R mutant, only K477R/E445Q showed further activity improvement. In the three-point and four-point mutants constructed based on K477R/E445Q, only K477R/E445Q/T88S/F102V achieved a higher relative yield, whereas the activity of K477R/E445Q/T88S was even lower than the wild type. This result suggested that the combination of positive mutations did not necessarily lead to an additive effect. In many cases, the mutations interact in a non-additive manner (Reetz 2013; Yu and Dalby 2018). Non-additivity could not only occur in the neighboring mutations but also between distant mutations through a network of interactions (Whitley and Lee 2009; Reetz et al. 2009), the mechanism of which is yet to be explored.

**Table 1:** Cadaverine yield of CadA multi-site mutants relative to the wild type

Mutants	Relative yield (%)
Wild Type	100
T88S	105.2
K477R/E445Q	124.1
K477R/F102V	103.2
K477R/T88S	105.5
K477R/E445Q/T88S	93.4
K477R/E445Q/F102V	103.5
K477R/E445Q/T88S/F102V	137.7

### Effect of pH and temperature on the activities of CadA and its mutants

**Fig. 4:**
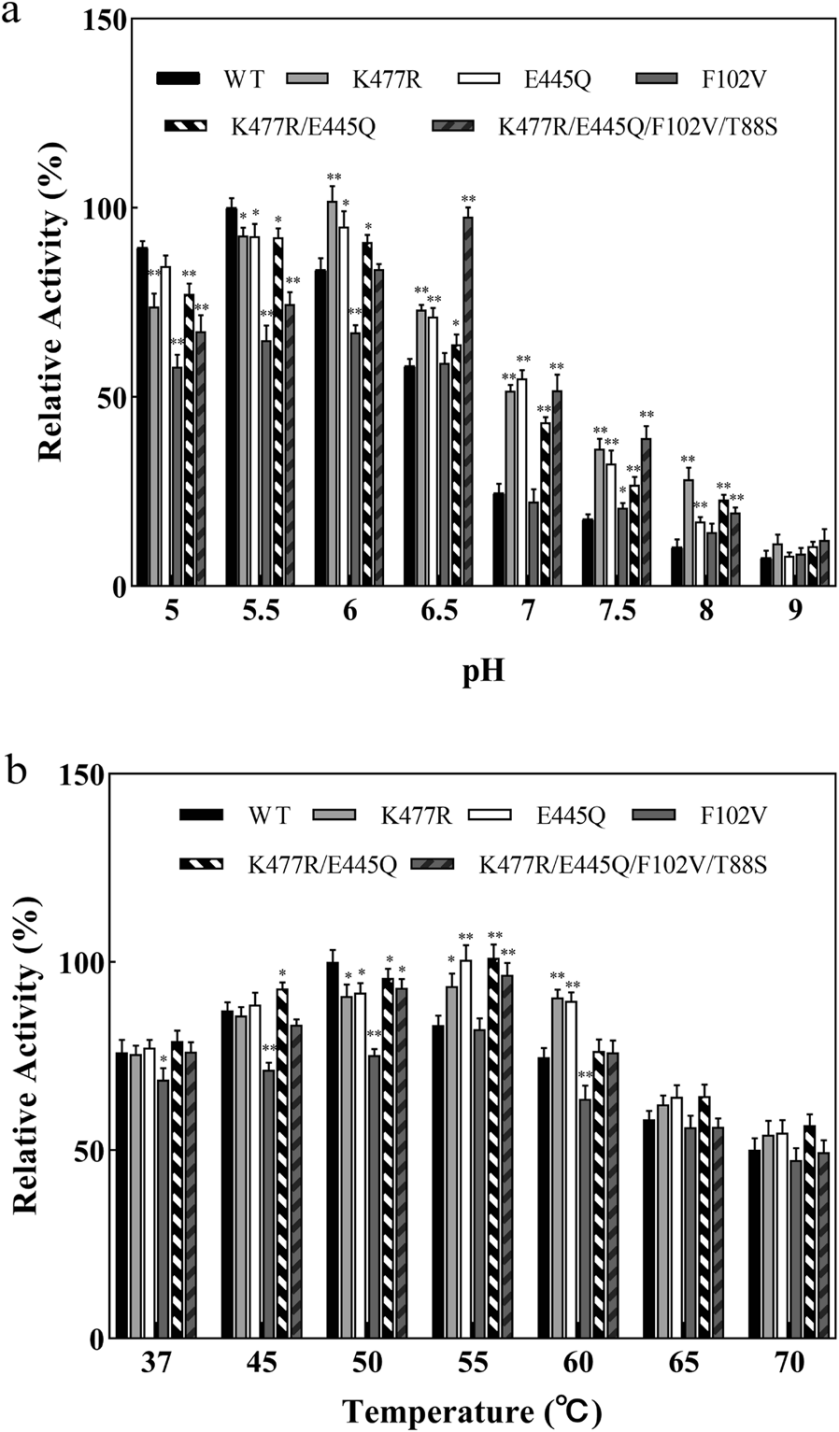
Effect of pH and temperature on the activities of CadA and its mutants. **a** Relative activities of wild-type CadA and its mutants at different pH. All the relative activities were calculated based on the activity of the wild-type CadA at pH 5.5. **b** Relative activities of wild-type CadA and its mutants at different temperatures. All the relative activities were calculated based on the activity of the wild-type CadA at 50 ºC. The error bars represent standard deviations calculated from triplicate experiments, and statistical significance of the different relative cadaverine yield in comparison with the wild type was evaluated using Student’s *t* test (*, *P* $<$ 0.05, **, *P* $<$ 0.01)

The optimal pH of the wild-type CadA and its mutants was determined by measuring the enzyme activities at pH 5.5–9.0 (Fig. 4a). The optimal pH of CadA_WT was 5.5, and its enzyme activities were dramatically decreased with the increase of pH. The mutants showed similar decreasing trends in the activity at alkaline conditions, although the activities were higher than those of CadA_WT in alkaline conditions (pH 7.5–9.0). Most of the mutants had higher optimal pH than CadA_WT. The pH optimum of the best mutant K477R/E445Q/T88S/F102V reached 6.5, while the three single-point mutants (K477R, E445Q, and F102V) had a pH optimum of 6.0. The double mutant K477R/E445Q had an optimal pH range of 5.5–6.0. Meanwhile, the enzyme activities of CadA_WT and its mutants were measured at different temperatures ranging from 37 to 70 ºC (Fig. 4b). The optimal temperature of all mutants was found to be 55 ºC, which was 5 ºC higher than that of CadA_WT (50 ºC).

### Kinetic analysis of CadA and its mutants

The kinetic parameters of the wild-type CadA and its mutants were analyzed by measuring the initial velocities over the substrate concentration range of 0.4–8.0 mM at pH 5.5 and 37 ºC, as well as at pH 8.0 and 50 ºC. In general, the *K*_m_ values were increased and the *K*_cat_/*K*_m_ values were decreased for all enzymes at pH 8.0 and 50 ºC. Nevertheless, similar trends were observed in the kinetic parameters under these different conditions. As shown in Table 2, the enhanced catalytic efficiency (*K*_cat_/*K*_m_) of mutants K477R, K447R/E445Q, and K477R/E445Q/T88S/F102V mainly originated from the decreased *K*_*m*_. This result indicated that the K477R mutation may increase the affinity of the enzyme to the substrate. For E445Q and F102V, the *K*_cat_/*K*_m_ values were either not obviously changed or decreased as compared to CadA_WT. The marginal changes in kinetic parameters for E445Q indicated that the E445Q mutation did not affect the active site of CadA, and the increase in cadaverine yield at high temperature and alkaline conditions mainly resulted from the improved stability. The lower *K*_cat_/*K*_m_ value of F102V than CadA_WT at pH 5.5 and 37 ºC suggested that although the F102V mutation improved the stability of CadA, it may have a negative effect on its active site. Such a negative correlation between stability and enzyme activity is known as stability-activity trade-off, which is also reported in previous studies (Nagatani et al. 2007; Tokuriki et al. 2008).

**Table 2:** Kinetic parameters of the wild-type CadA and its mutants

mutant	pH 5.5 and 37 ºC			pH 8.0 and 50 ºC		
	*K*_m_ (mM)	*K*_cat_ (s^−1^)	*K*_cat_/*K*_m_ (s^−1^·mM^−1^)	*K*_m_ (mM)	*K*_cat_ (s^−1^)	*K*_cat_/*K*_m_ (s^−1^·mM^−1^)
WT	1.52 ± 0.15	81.09 ± 4.53	53.34	3.3 ± 0.69	73.75 ± 0.94	22.35
K477R	1.17 ± 0.25	83.59 ± 5.63	71.44	1.52 ± 0.17	79.26 ± 0.48	52.14
E445Q	1.44 ± 0.23	79.33 ± 3.79	55.09	2.64 ± 1.06	67.35 ± 1.68	25.51
F102V	1.66 ± 0.27	80.08 ± 6.32	48.24	3.01 ± 0.50	69.01 ± 0.89	22.92
K477R/E445Q	0.96 ± 0.21	83.53 ± 3.42	87.00	2.45 ± 0.25	71.69 ± 0.42	29.26
K477R/E445Q/F102V/T88S	1.09 ± 0.63	83.66 ± 4.67	76.75	1.78 ± 0.78	82.76 ± 3.67	46.50

Kinetic measurement at various lysine concentrations (0.4–8 mM) were performed at pH 5.5 and 37 ºC, or at pH 8.0 and 50 ºC in sodium acetate buffer solution. The results are presented as means ± standard deviations for triplicate experiments

### pH and thermostability of CadA and its mutants

**Fig. 5:**
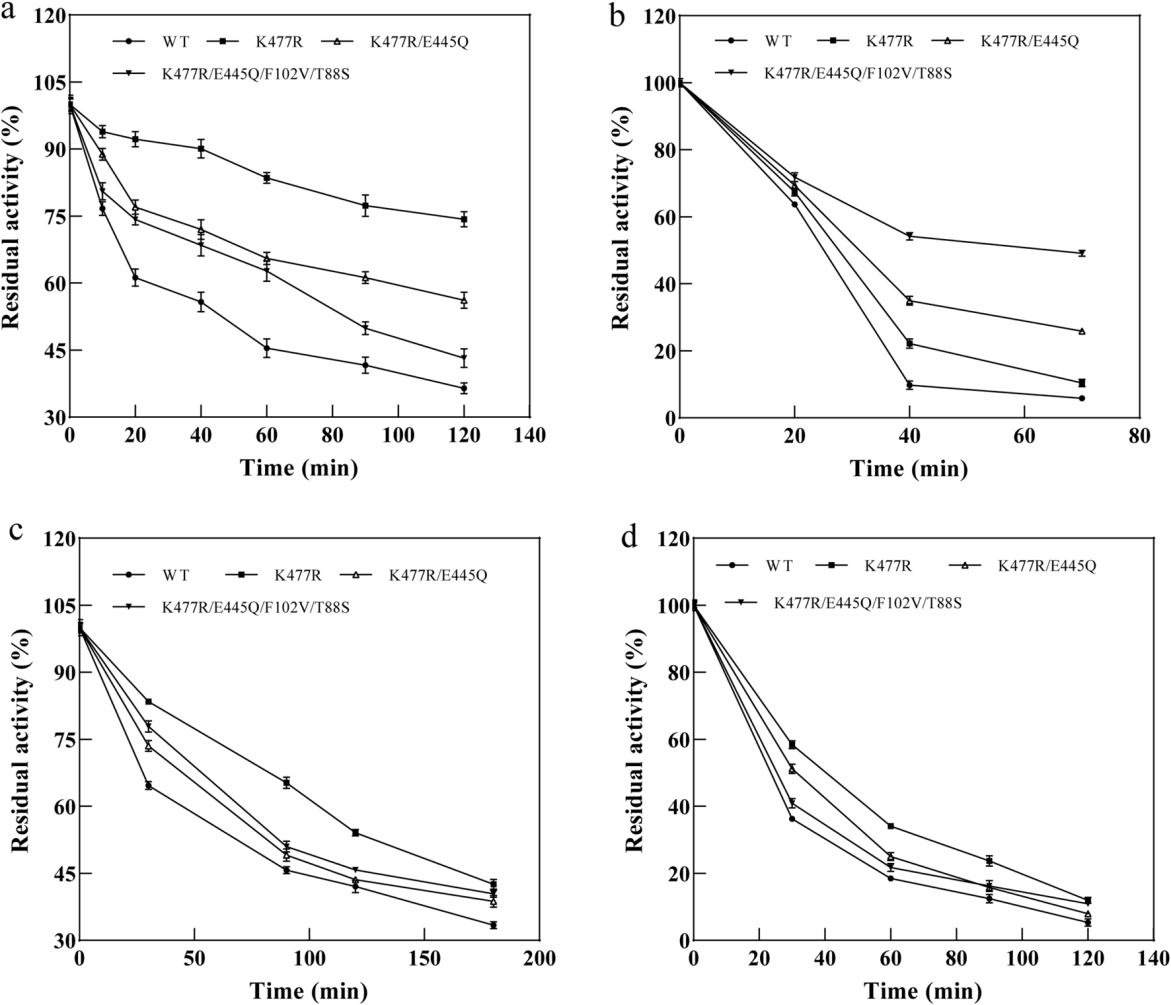
Thermostability and pH stability of the wild-type CadA and its mutants. **a** Residual activities of mutants and the wild-type CadA after incubation at 60 ºC. **b** Residual activities of mutants and the wild-type CadA after incubation at 70 ºC. **c** Residual activities of mutants and the wild-type CadA after incubation at pH 8.0 and 50 ºC. **d** Residual activities of mutants and the wild-type CadA after incubation at pH 9.0 and 50 ºC. The initial activities without incubation were set as 100%. The error bars represent standard deviations calculated from triplicate experiments

To evaluate the thermostability of K477R, K477R/E445Q, and K477R/E445Q/F102V/T88S, the residual activities of the variants after incubating at 60 ºC and 70 ºC were measured (Fig. 5a and Fig. 5b). The activity of the wild-type CadA and mutants decreased with increased incubation time. After 2 h incubation at 60 ºC, the wild-type CadA maintained 36.43% of activity, whereas the residual enzyme activities of mutants were higher (43.21–74.31%). In particular, the K477R mutant retained 74.31% of its initial activity. Likewise, after 70 min of incubation at 70 ºC, only 5.81% of activity remained for the wild-type CadA, while the best mutant K477R/E445Q/F102V/T88S maintained 49.14% of its initial activity. These results demonstrated the improved thermal stability of the mutants. The melting temperature (*T*_m_) of the purified proteins was also measured by circular dichroism (Chirascan plus, Applied Photophysics) (Additional file 1: Fig. S2). Compared to CadA_WT (66.7 ºC), all mutants had higher *T*_m_ values. To evaluate the pH stability of K477R, K477R/E445Q, and K477R/E445Q/F102V/T88S, the residual activities were measured after incubating under different pH (8.0 and 9.0) at 50 ºC (Fig. 5c and d). After 3 h incubation at pH 8.0, the wild-type CadA maintained 33.43% of its activity, whereas the mutants had higher residual activities (38.75–42.57%). When incubated at pH 9.0 for 2 h, only 5.32% of activity remained for the wild-type CadA, while the residual activities were 11.95%, 7.98%, and 10.92% for K477R, K477R/E445Q, and K477R/E445Q/F102V/T88S, respectively. These results suggested that the pH stability was enhanced for the mutants.

### Molecular dynamics simulations of CadA_WT and its mutants

To explore the mechanism behind the stability improvement of the mutants, molecular dynamics simulations were conducted. Considering the correlation of protein stability and the rigidity of its structure, RMSF (Root Mean Square Fluctuation) analysis of CadA_WT and its mutants was conducted. RMSF indicates the magnitude of change of each atom relative to its average position and has a mathematical relationship with the B-factor (1). As an important indicator of the rigidity of the protein structure, the larger the value of B-factor, the greater the flexibility (Parthasarathy and Murthy 2000; Sun et al. 2019). Increasing the rigidity of local areas in the protein has been shown to improve the stability of the whole protein (Wijma et al. 2013), and rigidifying flexibility sites (RFS) has been demonstrated as an effective approach for increasing protein stability (Yu and Huang 2014).

$$\mathrm{B}-\mathrm{factor}={\mathrm{RMSF}}^{2}\cdot \frac{8}{3}{\pi }^{2}. (1)$$

**Fig. 6:**
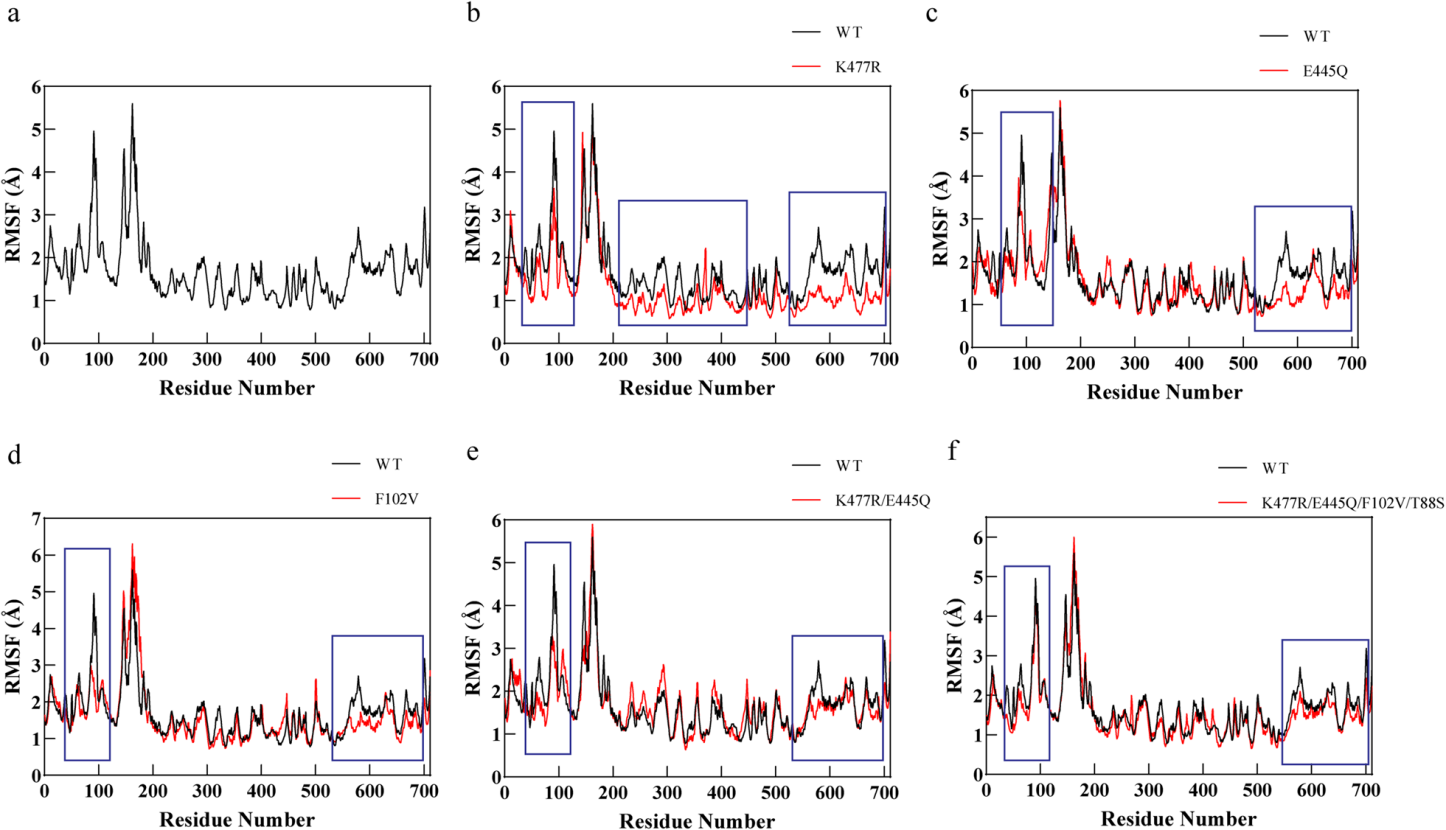
RMSF of each residue in the wild-type CadA and its mutants. **a** WT, **b** K477R, **c** E445Q, **d** F102V, **e** K477R/E445Q, **f** K477R/E445Q/T88S/F102V. The black solid lines indicate the wild type; the red solid lines indicate the mutants. The blue boxes indicate the areas where the RMSF values of the mutants have decreased

In comparison to CadA_WT, all mutants had 2 or more regional structures with lower RMSF, indicating higher rigidity (Fig. 6). Therefore, the improved rigidity of regional structures may contribute to the overall stability of the CadA at high temperatures and alkaline pH conditions. Moreover, these improved rigid regions are mostly found in the C- and N-terminal of CadA. In 3D structure models, these regions are mainly located at the surface and the decamer interface of CadA. This result implied that improving the rigidity of the decamer interface and surface of CadA may both affect its stability. The changes in RMSF values of these distant regions also implied a complex relationship between the introduction of mutation sites and the RMSF values of each amino acid residue in the protein (Yu and Dalby 2018).

It has been reported that introducing hydrogen bonds (Akbulut et al. 2013), salt bridges (Wu et al. 2015), β-folding, and charged residues exposed to solvents (Gribenko et al. 2009) can improve the enzyme tolerance to extreme environments, such as high temperature, strong acid, and strong alkali. Sáez-Jiménez et al. (Sáez-Jiménez et al. 2015) improved the acid stability of versatile peroxidase by directed evolution, finding the new salt bridges in the mutant as the main reason for the enhanced pH stability. Yokot et al. (2006) found that there were more salt bridges and polar amino acids in thermophilic proteins. Since the inactivation of CadA during the reaction is caused by dissociation of the decamer to form dimers under alkaline conditions, the interactions at the subunit interface are of great importance to its stability. The main interactions at the subunit interface are salt bridges and hydrogen bonds in the wild-type CadA (http://www.ebi.ac.uk/pdbsum/). Thus, salt bridge and hydrogen bond analysis of CadA_WT and its mutants might shed light on the mechanism behind the stability improvement of the mutants.

**Fig. 7:**
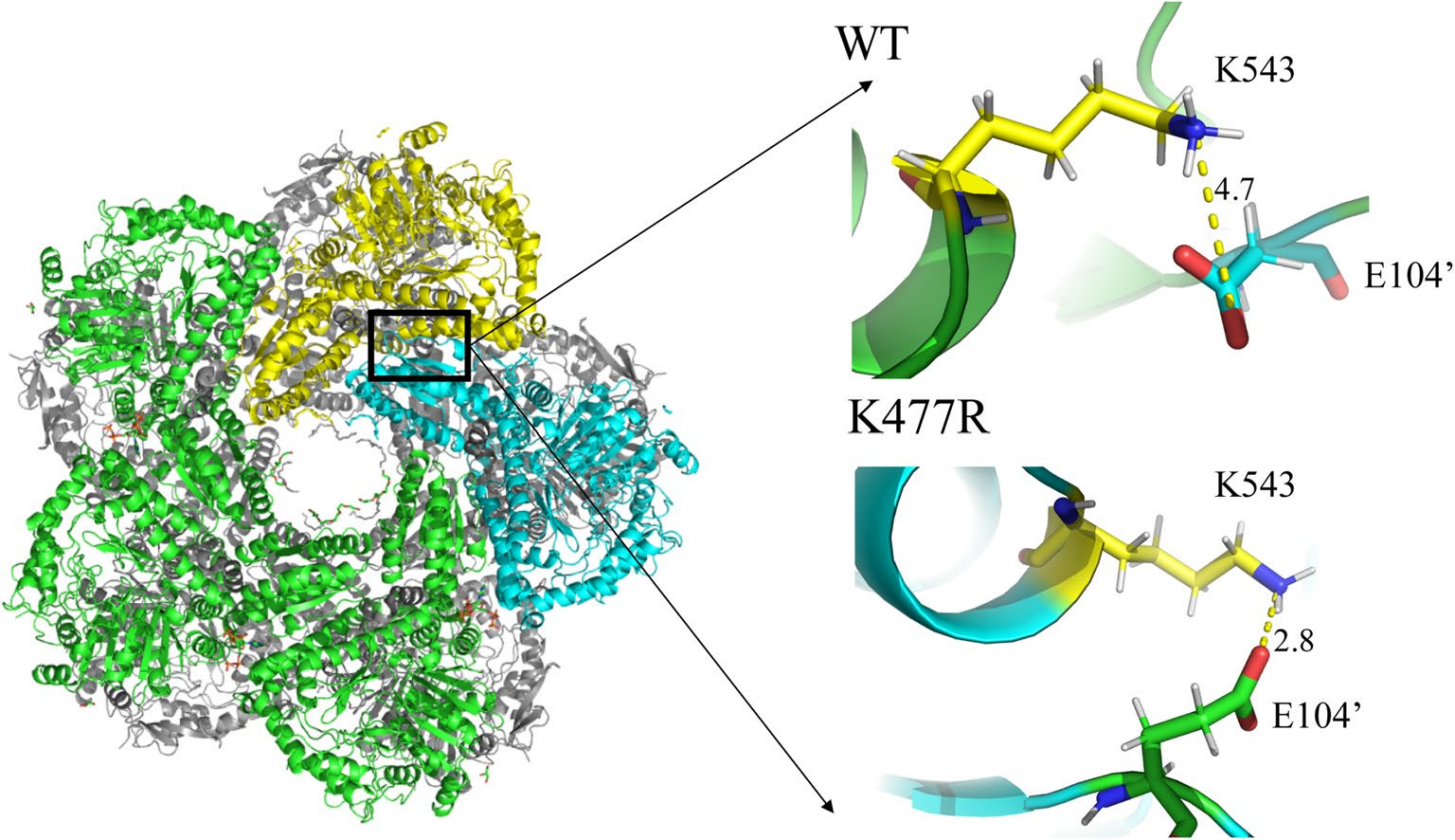
Salt bridge between K543 and E104’ at the decamer interface of CadA. The minimum energy conformation was extracted by Amber cpptraj, and the distance of the interacting residues was calculated by pymol

Analysis of molecular dynamics trajectory showed a higher number of salt bridges with a formation probability of more than 80% in all the five mutants as compared to CadA_WT (Table 3 and Additional file 1: Tables S3–S8). This result indicated the improved stability of these mutants might be partially due to the increased number of salt bridges. Most of the salt bridges are located on the surface of CadA, and only a pair of salt bridge (K543-E104’) is located on the decamer interface. Noticeably, introduction of the mutation sites changed the formation probability of salt bridge between K543 and E104’. Particularly, in mutant K477R, the distance between the NH_3_^+^ of K543 and the COO^−^ of E104' was shorter than 3.5 Å during most time of the 1000 ps molecular dynamics trajectory (Additional file 1: Fig. S3), and the probability of forming a salt bridge was greatly increased from 7.9 to 88.2% (Fig. 7). Strengthening the salt-bridge interactions at the subunit interface in K477R may prevent CadA from dissociating under alkaline conditions. In other single-point mutants E445Q and F102V, the probability of forming a salt bridge between K543 and E104’ was 9% and 2.8%, respectively, and there was no significant change in the salt-bridge interactions at the subunit interface. In the multi-site mutants K477R/E445Q and K477R/E445Q/F102V/T88S, the formation probability of K543-E104’ salt bridge was 54.4% and 5.9%, respectively. These results implied that complex interactions might occur when several individual mutations were combined and that designing new salt bridge pairs in the surface of CadA may be an effective measure to obtain high-stability variants.

**Table 3:** Number of salt bridges in the wild-type CadA and its mutants

Mutants	Total number of salt bridges (probability > 5%)	Number of salt bridges (probability > 80%)
WT	28	7
K477R	25	10
E445Q	29	8
F102V	28	9
K477R/E445Q	30	8
K477R/E445Q/T88S/F102V	28	8

As for hydrogen bonds, no new hydrogen bonds were formed at the subunit interface of all five mutants, but the formation probability of some existing hydrogen bonds was increased compared to the wild-type CadA (Table 4). The enhancement of hydrogen-bonding interactions at the decamer interface may contribute to the improved stability of mutants. Noticeably, the K477R mutation affected both the salt-bridge and hydrogen-bonding interactions at the subunit interface although it is not located there. This result implied that there are long-range interactions in CadA, which may be mediated by a complex network of interactions. Exploring the mechanism of such long-range interactions would provide valuable guidance for future protein modification.

**Table 4:** Probability of hydrogen bonds formation at the decamer interface of the wild-type CadA and its mutants

Hydrogen bonds	WT	K477R	E445Q	F102V	K477R/ E445Q	K477R/E445Q/F102V/T88S
GLN32-GLU21’	28.6	63.2	7.5	0.2	3.7	0.3
ASN49-LEU107’	30.6	1.7	1.2	58.7	3.5	41
LYS434-SER87’	6.5	46.8	29.5	94.7	34.9	0.9
ARG441-LEU89’	39	79.3	87.7	71	63.4	83.3
ARG543-GLU104’	48.6	73	64.4	32.2	58.3	48.9
ARG551-LEU93’	31.3	29.4	16.3	7.4	36.2	36
ARG551-LEU96’	62.3	76.6	94.3	27.6	91.2	52.1

**Fig. 8:**
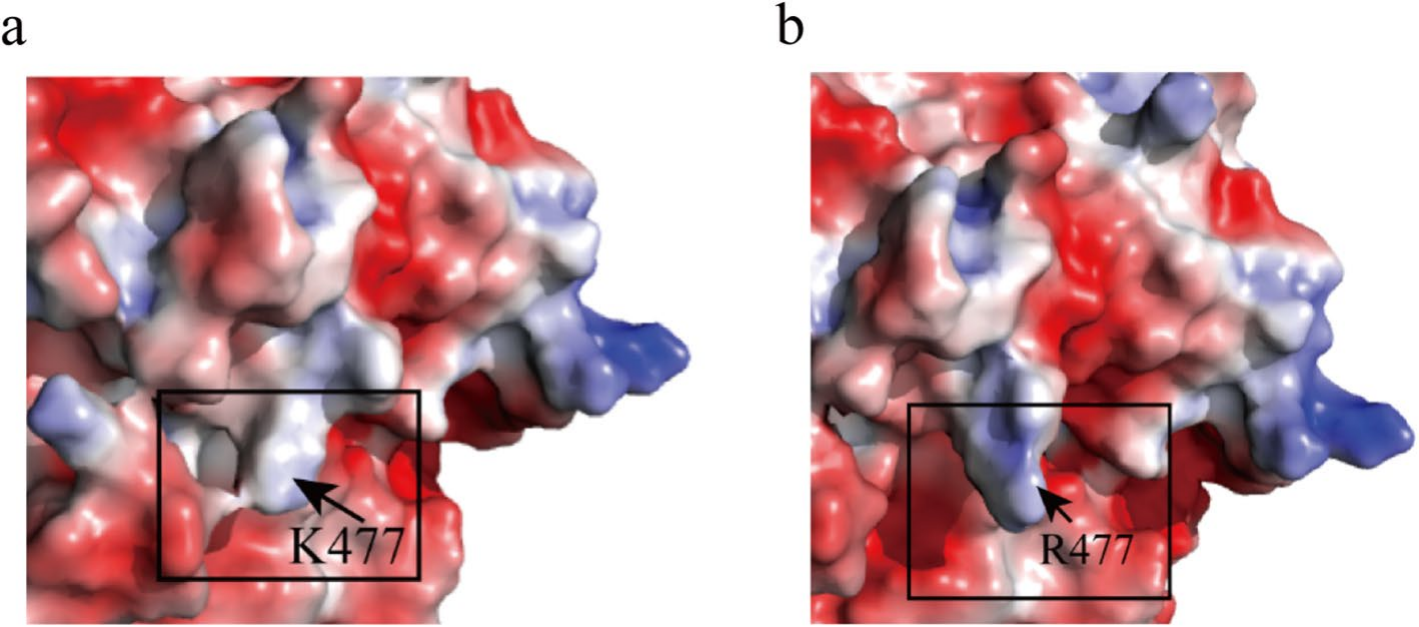
Comparison of the electrostatic surface around residue 477. **a** The wild-type CadA. **b** The K477R mutant. Blue means positive charge, while red means negative charge

Considering the surface location of the 477 site, its mutation alone or in combination with others may cause changes in the charge on the protein surface. Previous studies had found that optimizing the charge distribution on the protein surface and eliminating adverse electrostatic forces could enhance the protein stability (Eijsink et al. 2004; Strickler et al. 2006), and that removal of the negative charge contributed to the improved stability of ω-transaminases (Meng et al. 2020). Comparison of the surface charge between the wild type and the K477R mutant showed that the K477R mutation led to more positive charges around the 477 site (Fig. 8). The positive charge may neutralize the negative charge on the protein surface and reduce unfavorable electrostatic interactions on the protein surface, which may partially contribute to the improved stability.

### Evaluation of wild-type CadA and the K477R/E445Q/F102V/T88S mutant for cadaverine production

**Fig. 9:**
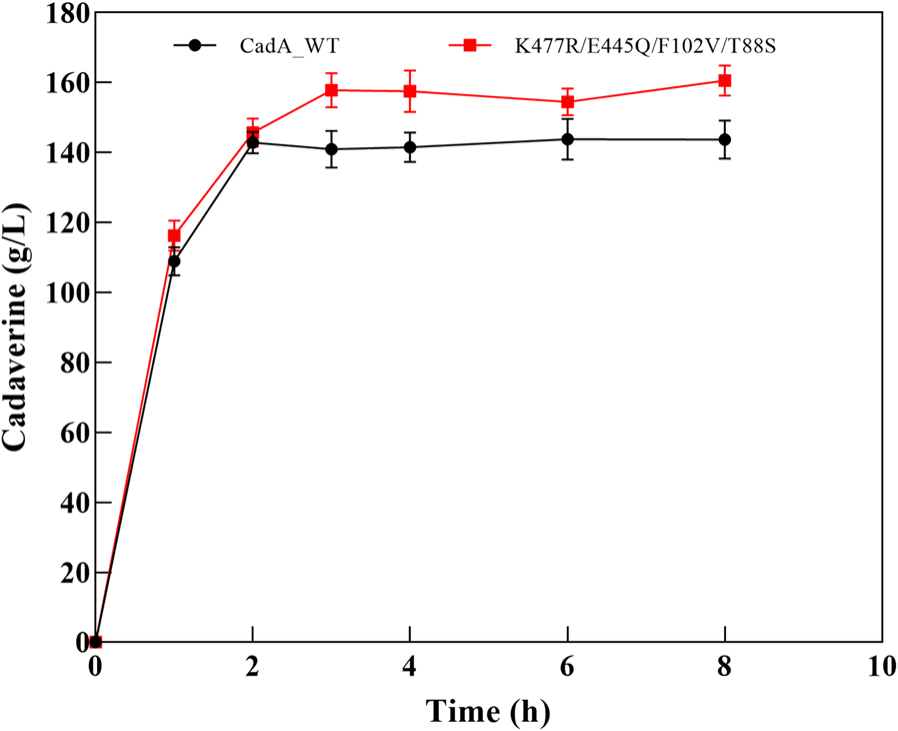
Cadaverine production by whole-cell catalyst of the wild-type and mutant CadA. The reaction was conducted using 2.0 M lysine hydrochloride as the substrate and at an initial pH of 6.0 and 50 ºC, and the pH was not regulated during the reaction. The error bars represent standard deviations calculated from triplicate experiments

The catalytic performance of the best mutant K477R/E445Q/F102V/T88S was compared with the wild-type CadA in whole-cell reactions with 2.0 M lysine hydrochloride as the substrate. The reaction was conducted without pH regulation at an initial pH of 6.0 and 50 ºC. After 8 h, the wild-type CadA produced 143.7 g/L cadaverine with a 70% conversion rate, whereas the mutant K477R/E445Q/F102V/T88S produced 160.7 g/L cadaverine with a 78.5% conversion rate. Compared to the wild-type CadA, mutant K477R/E445Q/F102V/T88S reached the platform period later (Fig. 9). The improvement of cadaverine yield was the result of the improved thermal and alkaline stability and also the increased *K*_cat_ value of this mutant. In a previous study, pH-unregulated L-lysine bioconversion mediated by a CadA mutant F14C/K44C/L7M/N8G obtained by rational design delivered 157 g/L cadaverine from 2.0 M lysine after 9.5 h of reaction (Hong et al. 2017). However, 10% v/v of cell extract was used as the biocatalyst in that study, and preparation of cell extract would lead to extra costs as compared to whole cells. Besides protein engineering, immobilization is another efficient method to improve the stability of enzymes (Kumar 2020; Bayramoglu et al. 2020). Immobilization of wild-type CadA had been reported to improve cadaverine production as compared to the free enzymes (Bhatia et al. 2015; Zhou et al. 2020). If we could immobilize K477R/E445Q/F102V/T88S in the future, the catalytic stability may be further enhanced.

## Conclusion

In this study, the thermal and alkaline stability of the *E. coli* lysine decarboxylase CadA was improved by combining directed evolution and computation-guided virtual screening. Interestingly, site 477 residue located at the protein surface and not the decamer interface was found as an engineering target in directed evolution, and its mutation from lysine (K) to arginine (R) increased both the affinity of the enzyme to the substrate and the formation probability of salt bridges and hydrogen bonds at the decamer interface. The final four-point mutant K477R/E445Q/F102V/T88S displayed a superior catalytic property (up to 37.7% improvement in cadaverine yield) than the wild-type CadA with both increased thermal and alkaline stability (residual activity 10.92% vs. 5.32% after 2 h incubation at pH 9.0, residual activity 49.14% vs. 5.81% after 70 min incubation at 70 ºC) and *K*_cat_/*K*_m_ value (76.75 vs. 53.34 s^−1^·mM^−1^ at 37 ºC, pH 5.5). Higher cadaverine was produced by the mutant K477R/E445Q/F102V/T88S from 2.0 M lysine hydrochloride at 50 ºC and a pH-unregulated condition than the wild-type CadA (160.7 g/L vs. 143.7 g/L). The pH-unregulated whole-cell reaction mediated by the CadA mutant K477R/E445Q/F102V/T88S can reduce the amount of acid and alkali required in whole-cell catalysis for cadaverine production. Meanwhile, these results demonstrated that directed evolution and computation-guided virtual screening is a useful and powerful means to generate enzymes with superior catalytic performances. In addition, the findings that mutations in sites that are located on the surface of CadA also influenced its stability, and that the improved stability was the result of the improved rigidity of regional structures, increased number of salt bridges and enhancement of hydrogen bonds at the multimeric interface, would provide a useful reference for stability improvement of other proteins.

### Supplementary Information

**Additional file 1.** Figures S1–S3 and Table S1–S8.

## Data Availability

Not applicable.

## Abbreviations

CadA: L-lysine decarboxylase
PLP: Pyridoxal-5-phosphate
MEGAWHOP: Megaprimer PCR of whole plasmid
CUPSAT: Cologne University Protein Stability Analysis Tool
DEEMM: Diethyl ethoxymethylenemalonate
TNBS: 2,4,6-Trinitrobenzenesulfonic acid
HTS: High-throughput screening
*T*_m_: The melting temperature
DCS: Differential scanning calorimetry
RMSF: Root Mean Square Fluctuation
DTT: Dithiothreitol
TNP-cadaverine: N,N’-bisnitrophenylcadaverine
