# Supplementary material for: Enhanced thermal and alkaline stability of L-lysine decarboxylase CadA by combining directed evolution and computation-guided virtual screening
Source: Bioresour Bioprocess. 2022 Mar 21;9(1):24. doi: 10.1186/s40643-022-00510-w (PMC10992825; doi:10.1186/s40643-022-00510-w)
Supplement: Supplementary file 1 — Additional file 1. Figures S1–S3 and Table S1–S8. [file 40643_2022_510_MOESM1_ESM.pdf]

Enhanced thermal and alkaline stability of L-lysine decarboxylase CadA by combining directed evolution and computation-guided virtual screening

Yang Xi[a], Lidan Ye[a,b*], Hongwei Yu[a*]

[a] Institute of Bioengineering, College of Chemical and Biological Engineering, Zhejiang University, Hangzhou 310027, China

[b] Hangzhou Global Scientific and Technological Innovation Center, Zhejiang University, Hangzhou 311200, China

* Corresponding authors:

Lidan Ye: yelidan@zju.edu.cn

Hongwei Yu: yuhongwei@zju.edu.cn

Supplementary information

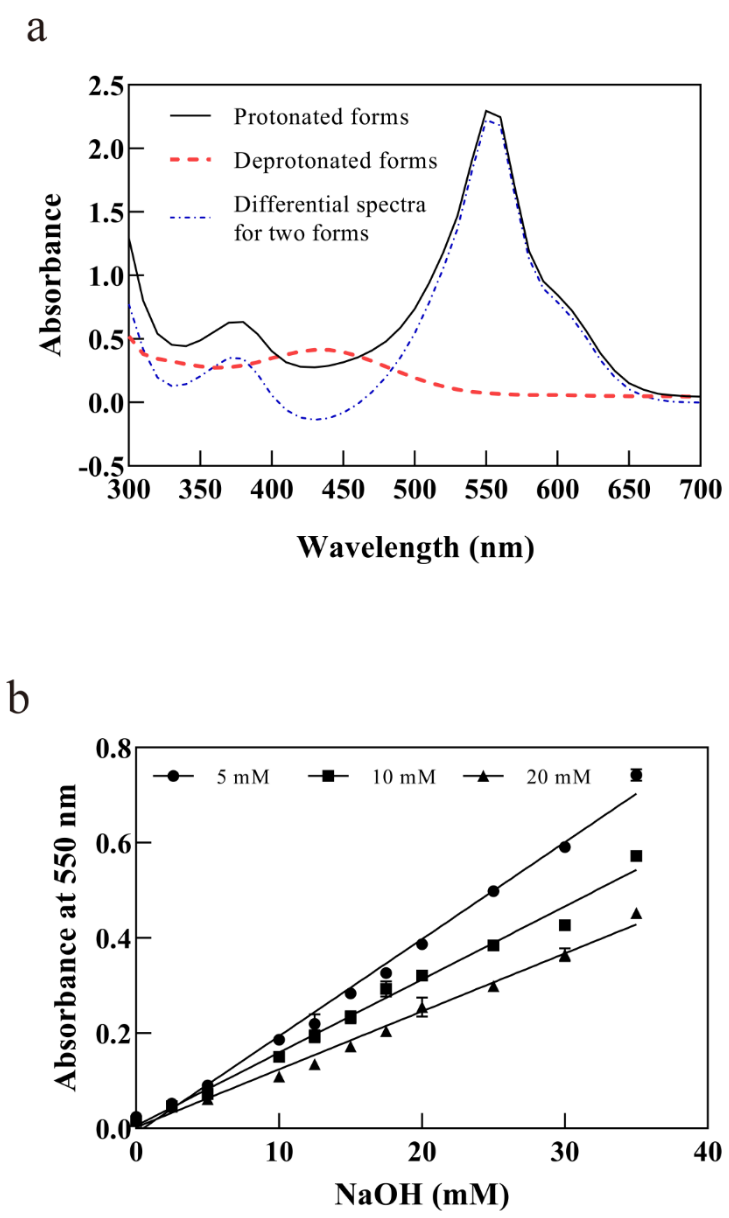

Fig. S1 Establishment of the HTS method. a. Absorption spectra of the mixed indicator in protonated and deprotonated forms. b. Effect of buffer concentrations on the absorbance at 550 nm of the mixed indicator. The assay mixtures contained 100 mM L-lysine hydrochloride, 0.1 mM PLP, pH 8.0 borate buffer at 5 mM (■),10 mM (♦), 20 mM (▲), respectively, and 10 μL mixed indicator. The error bars represent standard deviations calculated from triplicate experiments

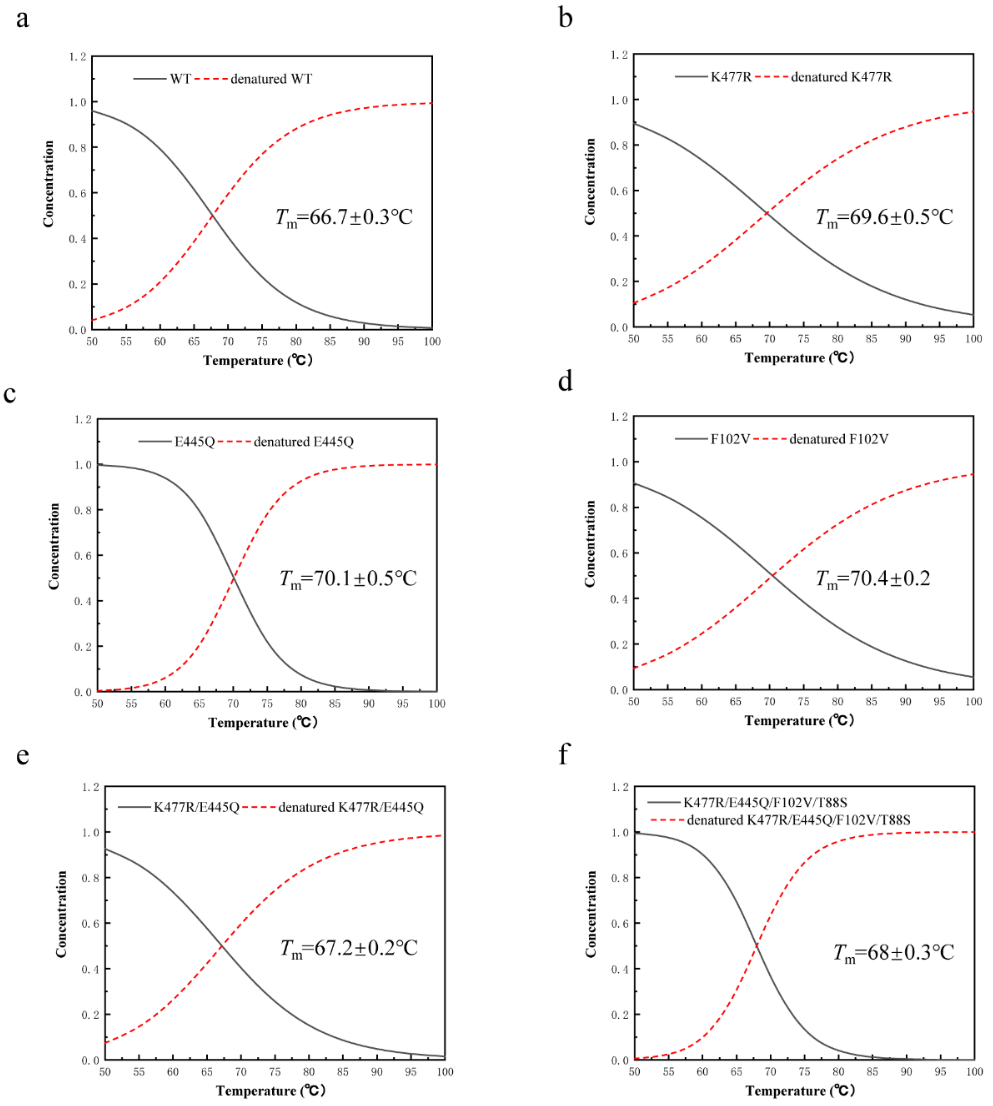

Fig. S2. Thermal stability of the wild-type CadA and its mutants. a. T_m of the wild-type CadA; b. T_m of K477R; c. T_m of E445Q; d. T_m of F102V; e. T_m of K477R/E445Q; f. T_m of K477R/E445Q/F102V/T88S.

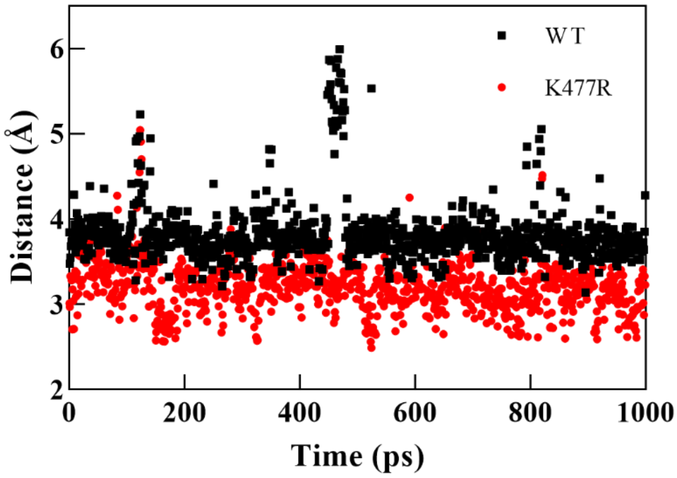

Fig. S3 Distance between the nitrogen of NH_3^+ of K543 and the oxygen of COO^- of E104' in the wild-type CadA and the K477R mutant

Table S1. Primers used for error-prone PCR

Primers	Sequence (5'-3')
cadA-F	CGGATCCATGAACGTTATTGCAATATTG
cadA-R	CCGCAAGCTTTTTTTTGCTTTCTTCTTTC

Table S2 Computational saturation mutagenesis in E445, T544, F102 and D41

Mutation	iStable	Cupsat	Rosetta ddG kJ/(mol·subunit)
E445A	×	×	-3.49
E445V	×	√	-0.59
E445L	×	√	11.49
E445I	×	√	7.22
E445F	×	√	-3.12
E445W	√	√	20.97
E445M	×	√	11.19
E445P	×	√	17.27
E445G	×	×	-0.18
E445S	√	×	9.86
E445T	√	×	8.26
T544G	×	×	13.72
T544H	×	×	10.51
T544I	√	√	-1.47
T544M	×	√	-6.12
T544V	×	√	17.09
T544K	×	√	6.71
T544Q	×	√	13.89
T544L	×	√	-6.17
T544P	×	×	10.11
T544W	×	×	5.01
T544S	×	×	-29.10
T544Y	×	×	-0.02
T544N	×	×	-12.26
T544R	×	×	6.78
D41A	√	×	-0.69
D41M	√	√	7.86
D41W	×	√	9.97
D41F	×	√	11.65
D41Y	×	√	14.43
D41E	√	×	9.44
D41H	×	×	12.70
D41V	×	×	0.77
D41R	√	×	7.10
D41K	×	×	24.66
D41Q	×	√	7.74
D41N	×	×	17.74
D41T	×	×	2.29
D41S	×	×	9.37
D41G	×	×	13.26

D41P	×	×	26.26
D41I	×	×	-1.60
D41L	√	×	14.2
F102A	×	√	20.14
F102V	√	√	7.51
F102L	√	√	4.51
F102I	√	√	6.40
F102W	×	√	5.32
F102M	×	×	19.71
F102P	×	×	20.38
F102G	×	×	12.97
F102S	×	×	19.56
F102T	×	√	5.38
F102Y	√	×	1.32
F102N	×	×	9.26
F102Q	×	×	9.52
F102H	×	×	33.71
F102K	×	×	20.36
F102R	×	×	19.04
F102D	√	×	25.49
F102E	×	×	6.66

√: stability improvement; ×: stability decline

$ddG = \triangle G_{MUT} - \triangle G_{WT}$，$ddG < -1.0$ kJ/(mol·subunit): stability improvement

Table S3 Formation probability of salt bridges in the wild-type CadA

Salt bridge	Formation probability（%）
GLU627-LYS707	100
ASP58-LYS15	99.8
ASP309-LYS527	99.7
ASP452-LYS440	99
ASP561-LYS558	90.5
GLU11-ARG28	81.5
GLU663-ARG242	80.1
GLU279-ARG288	78.5
ASP316-LYS320	67.1
GLU667-ARG670	66.4
ASP501-LYS500	60.9
GLU201-ARG212	52.7
GLU582-LYS567	50.8
GLU69-LYS72	50.3
ASP122-LYS118	45.8
GLU438-ARG441	39.1
ASP555-ARG551	28.7
ASP122-ARG116	24.4
GLU47-ARG423	20.2
ASP542-LYS545	19.8
GLU142-LYS144	13.3
GLU633-LYS710	10
GLU68-LYS72	8.7
LYS543-GLU104’	7.9
ASP323-LYS325	7.1
ASP211-ARG353	6.3
ASP60-LYS15	5.9

GLU199-LYS198	5.8

Table S4 Formation probability of salt bridges in K477R

Salt bridge	Formation probability（%）
GLU627-LYS707	99.3
ASP58-LYS15	99.3
ASP452-LYS440	97.4
ASP309-LYS527	96.9
GLU582-LYS567	90.7
ASP561-LYS557	89.7
LYS543-GLU104’	88.2
GLU279-ARG288	86.6
ASP122-LYS118	85
GLU201-LYS198	81.1
GLU667-ARG670	70.7
GLU21-ARG24	67.6
GLU69-LYS72	62.7
GLU47-ARG423	53.7
GLU111-ARG28	44.7
ASP470-ARG468	39.2
ASP555-ARG551	24.1
GLU142-LYS144	21.1
ASP383-ARG353	19.8
GLU68-LYS72	16.8
ASP323-LYS325	12.2
ASP638-ARG703	10.6
GLU576-ARG575	7.9
GLU438-ARG441	7.6
GLU201-ARG212	7.1

Table S5 Formation probability of salt bridges in E445Q

Salt bridge	Formation probability（%）
ASP309-LYS527	100
ASP58-LYS15	100
GLU201-LYS198	99.1
ASP452-LYS440	98.5
GLU627-LYS707	97.5
ASP122-LYS118	93.9
ASP316-LYS320	83
ASP561-LYS557	82.6
ASP211-ARG353	76.9
GLU279-ARG288	74.3
GLU633-ARG242	70.2
GLU690-ARG698	64.5
GLU69-LYS72	53.1
GLU677-ARG670	46.4
GLU21-ARG24	45.6
GLU499-LYS500	43.4
GLU711-LYS626	27.1
ASP542-LYS545	22.9
GLU386-LYS198	18.5
ASP638-ARG703	18.5
GLU16-ARG20	17.6
GLU438-LYS437	13.4
ASP506-LYS595	13.4
GLU438-LYS434	11.5
ASP122-ARG141	10.9
LYS543-GLU104’	9
GLU482-ARG468	8.5

GLU576-ARG575	6.9
GLU438-ARG441	5.4

Table S6 Formation probability of salt bridges in F102V

Salt bridge	Formation probability（%）
ASP309-LYS527	100
ASP58-LYS15	98.8
ASP460-LYS437	98.5
ASP122-LYS118	96.4
ASP452-LYS440	93.9
GLU663-ARG242	89.7
GLU582-LYS567	87.8
GLU279-ARG288	86.6
GLU482-ARG468	82.1
GLU142-LYS138	76.9
GLU690-ARG698	74.6
GLU667-ARG670	64.3
GLU386-ARG212	61.4
ASP383-ARG353	61.4
GLU69-LYS72	56
ASP604-ARG608	45
GLU47-ARG423	36.2
ASP542-LYS545	36
GLU438-ARG441	33.7
GLU186-LYS138	32.5
ASP186-LYS557	32.3
ASP316-LYS320	30.5
ASP501-LYS500	26.6
GLU201-LYS198	15.5
GLU21-AGR24	11.4
GLU111-ARG28	7.6
GLU499-LYS500	7.1
GLU430-LYS434	5.4

Table S7 Formation probability of salt bridges in K477R/E445Q

Salt bridge	Formation probability（%）
GLU582-LYS567	99.6
ASP452-LYS440	99.3
ASP309-LYS527	98.7
ASP316-LYS320	95.7
GLU627-LYS707	94.8
ASP122-LYS118	94.2
GLU663-ARG242	91.3
ASP58-LYS15	88.2
GLU111-ARG28	79.2
GLU69-LYS72	78.7
GLU667-ARG670	73.6
ASP470-ARG468	69.9
GLU201-ARG212	67.3
GLU711-ARG643	66.3
GLU611-ARG263	59.7
GLU279-ARG288	59.5
LYS543-GLU104’	54.4
ASP638-ARG703	53
ASP383-ARG353	42
GLU438-ARG441	36.1
GLU386-ARG212	26.1
ASP604-ARG608	25.7
GLU430-LYS434	23.2
GLU690-ARG698	23
GLU633-LYS710	19.9
ASP542-LYS545	19.8
GLU68-LYS72	19.7

GLU47-ARG423	14.6
ASP457-LYS500	13.2
GLU142-LYS144	12.9

Table S8 Formation probability of salt bridges in K477R/E445Q/F102V/T88S

Salt bridge	Formation probability（%）
GLU627-LYS707	99.1
ASP309-LYS527	98.9
GLU201-LYS198	98.7
ASP561-LYS557	95.9
GLU47-LYS44	95.2
ASP316-LYS320	92.5
GLU582-LYS567	84.7
GLU673-ARG242	84.6
GLU279-ARG288	71.6
ASP470-ARG468	67.3
ASP122-LYS118	67.3
GLU667-ARG670	62.4
ASP383-ARG353	57.6
GLU386-ARG212	56.5
GLU111-ARG28	53.8
GLU588-ARG585	43.6
GLU438-LYS434	29.2
ASP542-LYS545	17.9
GLU69-LYS72	15.2
GLU611-ARG263	10.6
GLU21-ARG24	10.3
ASP40-ARG39	9
ASP604-ARG608	8
GLU68-LYS72	7.9
ASP555-ARG558	7.9
GLU142-LYS138	6.9
LYS543-GLU104’	5.9

GLU291-LYS287	5.5
